# The Importance of Adverse Childhood Experiences in Depressive Symptoms and Their Biological Consequences in Healthy Adults: Results of a Polish University Student Study

**DOI:** 10.3390/jcm12227093

**Published:** 2023-11-14

**Authors:** Joanna Rog, Michał Karakuła, Zuzanna Rząd, Aleksandra Fitowska, Agnieszka Brzezińska, Hanna Karakula-Juchnowicz

**Affiliations:** 1 Laboratory of Human Metabolism Research, Department of Dietetics, Institute of Human Nutrition Sciences, Warsaw University of Life Sciences (WULS-SGGW), 02-787 Warsaw, Poland; 2 Department of Analytical Chemistry, Medical University of Lublin, 20-950 Lublin, Poland; michalkarakula@gmail.com; 3 1st Department of Psychiatry, Psychotherapy and Early Intervention, Medical University of Lublin, 20-950 Lublin, Poland; zuzanna.rzad@umlub.pl (Z.R.); brzezinska2agnieszka@gmail.com (A.B.); hannakarakulajuchnowicz@umlub.pl (H.K.-J.); 4 Department of General and Coordination Chemistry and Crystallography, Institute of Chemical Sciences, Maria Curie-Sklodowska University, 20-950 Lublin, Poland; fitowskaola@gmail.com

**Keywords:** trauma, depression, adverse childhood experience, psychological stress, major depressive disorders, mental health, biomarkers, biochemical biomarkers, gut permeability, dysregulations

## Abstract

Adverse childhood experiences (ACEs) have a long-lasting effect on both physical and mental health. The aim of this study was to assess the consequences of ACEs and experienced stress on depression and the role of biological disturbances in this relationship in a student population. Potential participants filled out a screening questionnaire; 60 of 126 students met the inclusion criteria and were tested for the severity of stress and depressive symptoms, ACEs, dietary habits, and serum concentrations of biological markers. Depressive symptoms were related to a younger age (*p* = 0.012), a higher severity of stress (*p* = 0.001), ACEs (*p* = 0.007), and lower triglyceride (*p* = 0.01) and cortisol concentrations (*p* = 0.01). An inverse relationship between the triglyceride concentration and emotional abuse (R = −0.38) and emotional neglect (R = −0.33) was found. Occludin was positively associated with physical abuse (R = 0.31). Cortisol was inversely associated with emotional abuse (R = −0.35). Emotional neglect was associated with lipopolysaccharide binding protein (R = 0.38) and insulin levels (R = −0.31). The most promising multi-panel of biomarkers for recognizing mood symptoms included triglycerides, tight junction protein 1, and cortisol (cut-offs of ≤ 95.5 mg/dL, 0.72 ng/mL, and 134.63 ng/mL, respectively). This study confirmed the association between ACEs and depressive symptoms and the importance of psychological stress in developing mood disorders. ACEs could affect biological dysregulation. Some of the biological markers could be helpful in early detection of depression.

## 1. Introduction

The prevalence of common health problems among university students increased significantly during the COVID-19 pandemic [1,2,3]. Diseases that are more often observed in young individuals include depression, anxiety, and metabolic disturbances [4,5,6,7]. According to the results of a Polish study from 2022, approximately one in three students exhibit depressive symptoms [8]. This finding is consistent with a meta-analysis indicating that the prevalence of depressive symptoms in student populations is 34% [5]. Many studies have confirmed that lifestyle factors are key to maintaining well-being and metabolic health [9,10]. Mood disorders and metabolic disruptions may share common pathological synergistic mechanisms, including the interplay between the loss of gut barrier integrity and inflammation [11]. These processes are closely related to lifestyle factors, such as dietary habits, physical activity, and exposure to psychological stress [12,13,14]. Among medical and health science students, certain lifestyle behaviours (including insufficient physical activity and sleep, problems with maintaining a stable body weight, and using alcohol) have been found to be linked to poor mental health [8]. In a group of young Polish men, healthy dietary patterns and regular physical activity were related to a reduced risk of adiposity and metabolic abnormalities, including abnormal levels of blood glucose, triglycerides, and cholesterol and hypertension [15]. 

The consequences of depression go beyond patients’ functioning and quality of life and extend to somatic health. Depressive symptoms are longitudinally associated with hypercholesterolemia, diabetes, and erectile dysfunction. During acute episodes of depression, insulin resistance is increased, and insulin has been proposed as a biomarker of the a metabolic subtype of depression [16]. Metabolic, inflammatory, and HPA-axis dysregulations are proposed as components of depression. However, the exact mechanisms of the disruptions are unclear [17].

One systematic review showed that individuals with severe mental illness and a history of adverse childhood experiences (ACEs) are more likely to demonstrate metabolic syndrome than individuals without ACEs [18]. Patients with major depressive disorder (MDD) showed neurometabolic changes, and ACEs modified these changes, leading to longer and more episodes of depression [19]. An overall self-reported worldwide prevalence of ACEs is estimated at 36.3% [20]. ACEs have a great impact on mental health. According to a meta-analysis, ACEs are associated with a two-fold higher odds of anxiety disorders, internalizing disorders, depression, and suicidality [21]. It has been shown that ACEs increase the risk of depressive symptoms in a dose–response relationship [22].

ACEs also have profound and long-lasting effects on physical health. This negative impact on health outcomes may be explored by dysregulation of the hypothalamic-pituitary-adrenal (HPA) axis and contributions to pro-inflammatory cascade events [23]. Cortisol is the most used physiological marker of stress and HPA-axis activation. Cortisol is strongly related to metabolic dysregulation and is involved in changes in glucose and lipid metabolism [24]. The intestinal is a well-known organ for modifying metabolic response and mental status [14]. The human body has many mechanisms for maintaining gut barrier homeostasis. However, being overwhelmed by adverse childhood and life experiences, improper dietary patterns, and other unhealthy lifestyle choices may contribute to a higher risk of a comprehensive range of health disorders [14]. 

Despite the confirmed relationship between ACEs and mood disorder symptoms, the number of studies examining the role of biological processes in this relationship is limited. Biomarkers would be better indicators of psychological symptoms and mental health compared to the most often used interviews and questionnaires. There is no single mental health-specific biomarker. However, objective measurements of physiological processes allow us to detect subtle perturbations implicated in disease development [25]. This study aimed to assess the potential consequences of ACEs and experienced stress on depressive symptoms and the role of biological disturbances in this connection in populations of healthy individuals. The secondary aim was to establish a potential multi-panel of biomarkers for detecting mood disorder symptoms. 

The specific hypotheses of the study are as follows:

(1) The severity of ACEs is related to a greater severity of depression;
(2) ACEs affect biological processes, playing a role in mood symptom development and/or maintenance;
(3) Physiological stress is an additional factor explaining experiences of depressive symptoms.

## 2. Materials and Methods

### 2.1. Study Group

**Figure 1:**
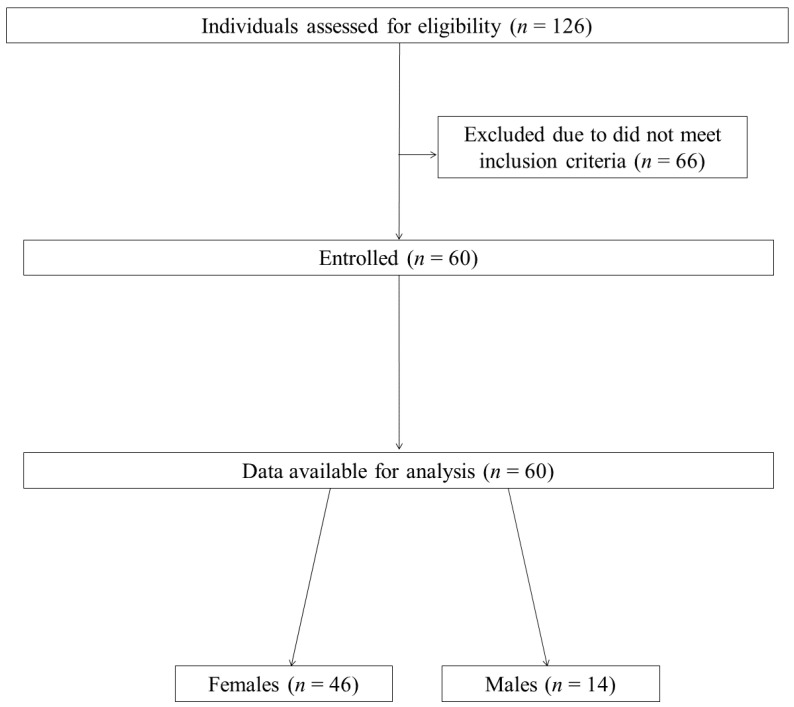
The flowchart of participant recruitment.

The group comprised students who volunteered to participate in the project. Recruitment and data collection occurred between July and September 2021. Before the examination, potential participants were asked to fill out a screening questionnaire to assess eligibility for the study via the Internet. In total, 126 individuals took part in the pre-examination. Of those, 60 met the inclusion criteria and none of the exclusion criteria. We included students of both sexes between 18 and 30 years old. The exclusion criteria of the project were as follows: suffering from psychiatric (Axis I, except nicotine abuse disorder), autoimmune, or cardiovascular diseases, inflammatory bowel disease, diabetes mellitus, or other diseases that, in the opinion of the researchers, affected metabolism or pro-/anti-inflammatory homeostasis. We excluded individuals taking any medications (except contraception). The participants who took non-steroidal anti-inflammatory drugs for one week, antibiotics for one month before the examination and with infection during entry to the project were also excluded. The information about clinical symptomatology was self-reported. The flowchart of participant recruitment is shown in Figure 1. The protocol of the study was approved by the Ethics Committee of the Medical University of Lublin (project ID: KE-0254/163/2021). All participants provided written informed consent.

### 2.2. Sociodemographic and Health-Related Data

Lifestyle and sociodemographic information was obtained via a fulfilled self-created survey. The questionnaire included questions about age, sex, number of years of education, field and year of study, chronic disease, and intake of medication and diet supplements. We asked participants about tobacco smoking, drinking alcohol, and relaxation techniques used.

### 2.3. Severity of Stress and Depressive Symptoms

The perceived stress intensity was assessed via the Polish adaptation of the Perceived Stress Symptoms Scale (PSS-10) [26,27]. This tool measures one’s own life situation-related stress over the last month. PSS-10 consists of 10 questions about subjective feelings related to personal problems and events, behaviours, and coping strategies. The answers are expressed on a scale from 0 to 4, and the overall scores range from 0 to 40. Higher results indicate greater stress severity. In this research, we used a cut-off point of 20 points to allocate participants to groups with high or low perceived stress [28].

The severity of depression/mood disorder symptoms was determined via the Beck Depression Inventory (BDI) [29]. The BDI is a self-reported questionnaire with 21 questions with four possible answer choices ranging from zero to three points. The overall scores range from 0 to 63. Higher scores indicate a more severe experience of symptoms. Like an earlier study, we established a cut-off point = 11 [30]. We chose a lower cut-off for depression to detect the mood symptoms experienced (no severe MDD) and establish early-stage biomarkers (before developing the full spectrum of symptoms) in a potentially healthy population.

### 2.4. Adverse Childhood Experiences

To assess ACEs, the Polish adaptation of a short form of the Childhood Trauma Questionnaire (CTQ) was used [31,32]. The scale consists of 28 items concerning experiences during childhood and adulthood divided into five subscales—physical abuse (PA), sexual abuse (SA), emotional abuse (EA), physical neglect (PN), and emotional neglect (EN)—with possible answer choices ranging from one to five points. Three items are designed to measure Minimisation/Denial (M/D); only the highest positive score (5) is counted. The overall scores range from 25 to 128.

### 2.5. Food Intake

Diet quality was assessed with Food Frequency Questionnaire 6 (FFQ-6), validated in the Polish population. The questionnaire contains questions about the usual consumption of 62 foods allocated to seven categories: sweeteners and snacks, milk products and eggs, cereal products, fats, fruits, vegetables and grains, meat products and fish, and drinks [33]. The frequency of consumption is expressed using six classes as follows: (1) never or almost never, (2) 1–3 days/week, (3) more than 3 days/week, (4) once a day, and (5) several times a day.

### 2.6. Blood Assessment

The blood samples were collected between 8 and 10 a.m. on the same day as other factors were examined. Approximately 20 mL of venous blood after overnight fasting was collected with S-Monovette (Sarstedt, Nümbrecht, Germany): serum gel (9 mL) and K3EDTA (2.7 mL) tubes were obtained. Assessment of blood and the triglyceride concentration was performed shortly after blood draw with an Accutrend^®^ Plus system glucometer (Cobas, Roche Farma, Barcelona, Spain) using whole-blood samples.

An enzyme-linked immunosorbent assay (ELISA) was applied to determine the concentration of markers. Serum was extracted via centrifugation at 2500× *g* for 10 min at room temperature. The samples were stored at −80 °C until further analysis immediately after pipetting into safe-lock Eppendorf tubes (Eppendorf^®^). We used the following ELISA kits:

- for gut barrier intestinal integrity assessment: Lipopolysaccharide Binding Protein (LBP; SEB406Hu, Cloud-Clone Corp., Wuhan, China), Occludin (OCLN; SEC228Hu, Cloud-Clone Corp., Wuhan, China), Tight Junction Protein 1 (TJP1, SEC262Hu, Cloud-Clone Corp., Wuhan, China), Anti-zonulin antibody (ZON-ab; E01A4280), histamine (E-EL-0032, BIOZOL, Eching, Germany), and Immunoglobulin G (IgG; E-EL-H0169, BIOZOL, Eching, Germany);
- for pro-/anti-inflammatory homeostasis assessment: Interleukin 1β (IL-1β; 850.006.096, Diaclone SAS, Besancon, France), Interleukin 10 (IL-10, 950.060.096, Diaclone SAS, Besancon Cedex France), and Tumour necrosis factor-α (TNF-α, 950.090.096, Diaclone SAS, Besancon, France);
- for metabolic function assessment: Insulin (10-1113-01, Mercodia, Uppsala, Sweden), Cortisol (CORT; DKO001, DiaMetra, Spello–Perugia, Italy), and Adiponectin (ADP; E-EL-H6122, BIOZOL, Eching, Germany).

### 2.7. Statistical Analysis

The statistical analysis was performed using Statistica 13 Software (StatSoft, Inc., Tulsa, OK, USA). The distribution of continuous variables was determined via the Shapiro-Wilk test. We applied both parametric and non-parametric tests due to both Gaussian and non-Gaussian distribution of variables. A non-parametric test was also used for ordinary variables. Categorical variables were expressed as frequencies and percentages, and continuous variables as the mean and standard deviation (SD) (Gaussian distribution) or the median and maximum and minimum values (non-Gaussian distribution and ordinary variables). The chi-square, t-student and U-Mann–Whitney tests were applied to assess changes in the examined factors between two groups (depressed/non-depressed or stress/unstressed individuals). Pearson’s r and Spearman’s rho correlation values were calculated to determine the relationship between the examined factors in the population. The associations of depressive symptoms with the examined parameters were assessed by linear regression. A *p*-value of <0.05 was considered significant.

The classification tree model method (C&RT) was used to assess potential biological predictors helpful in recognizing depressive symptoms in a vulnerable population. C&RT is a data-mining method used to indicate variables with their cut-off value to categorize groups based on a specific future (e.g., presence/absence of symptoms or disease). Examining factors are split into two subset trees according to cut-off points during the classification. Each of the following subsets maximizes the homogeneity of the two resulting subgroups [34,35]. For C&RT results, the area under the receiver operating characteristic curve (AUC) with a 95% confidence interval (CI) for all models was calculated.

All questionnaires were previously validated for administration to the Polish population. For the measurement of internal reliability, Cronbach’s alpha for the tools was estimated. The Cronbach’s alpha coefficient calculated for our study was 0.77 for PSS-10, 0.89 for BDI, 0.73 for CTQ, and 0.84 for FFQ-6.

## 3. Results

### 3.1. Characteristics of the Examined Population

The characteristics of the examined students are shown in Table 1. Among 60 individuals, 23% (*n* = 14) were males. The median age was 23, and the median education time was 16 years. Most of the participants were in year V (*n* = 16, 27%) of their academic education, and most were medical students (*n* = 31, 52%). Nine participants (9%) were taking medication (hormonal contraception), and 26 of the students (43%) used supplements in their diet (mainly vitamin D). A quarter of the group (*n* = 16) smoked cigarettes, and 17 applied relaxation techniques (mainly yoga and meditation).

### 3.2. Characteristics of the Students’ Mental Health and Differences in Examined Factors Based on Mental Health Outcomes

Based on the PSS-10 results, 47 participants (78%) were vulnerable to psychological stress. Individuals with a higher severity of stress had more severe mood symptoms (*p* = 0.01) only but did not differ in biological markers or diet compared to students with no stress symptoms (*p* > 0.05). There were also no differences in age, education period, or CTQ points between students who did and did not experience stress. 

According to the BDI scale total points, 16 students (27%) experienced depressive symptoms. Mood disorder symptoms were related to a younger age (*p* = 0.012), a higher severity of stress (*p* = 0.001), ACEs (higher total CTQ score (*p* = 0.007), and the following subscales: emotional abuse (*p* = 0.001), emotional neglect (*p* = 0.005)), lower levels of triglycerides (*p* = 0.01) and cortisol (*p* = 0.01), and lower intake of meat and fish (*p* = 0.04).

### 3.3. Relationship between Mood Disorder Symptoms and Examined Variables

The BDI was positively associated with the PSS-10 total score (R = 0.36) and ACEs: total CTQ score (R = 0.34), emotional abuse (R = 0.37), emotional neglect (R = 0.40), and physical abuse (R = 0.32). The BDI score was inversely associated with age (R = −0.36) and education period (R = −0.37).

More depressive symptoms were negatively associated with meat and fish intake (R = −0.31), and physical abuse scores were positively associated with alcohol intake (R = 0.30). 

We found an inverse relationship between triglyceride concentrations and the following ACEs: emotional abuse (R = −0.38) and emotional neglect (R = −0.33). OCLN was positively associated with physical abuse (R = 0.31). Cortisol was inversely associated with emotional abuse (R = −0.35). LBP was positively associated with emotional neglect (R = 0.38). Insulin was negatively related to emotional neglect (R = −0.31).

### 3.4. Relationship between Mood Disorder Symptoms and ACEs

Linear regression analyses between the dependent variable (depressive symptoms defined as a BDI total score) and the independent variable (ACE defined as a CTQ total score) were performed. All analyses were adjusted for the following baseline covariates possibly affecting symptom severity (stress symptoms, education period, and cortisol levels).

We found a relationship between depressive symptoms and ACEs, education period, and stress symptoms even after adjusting for potential confounders (TGs and cortisol) (see Table 2). The indicated model explained approximately 29.23% of the variability in the total BDI score. After excluding cortisol as an additional factor, the model explained 26.04% of the depression variability, and the education period was statistically insignificant (*p* = 0.077). Another model that was adjusted for cortisol explained 33.83% of the variability in mood disorder symptoms.

### 3.5. The Potential Multi-Panel Biological Markers of Experiencing Symptoms of Depression 

A step in the direction of establishing possible biomarkers of MDD is the construction of pathway models for the prediction of mood symptom likelihoods in a vulnerable population. C&RT analysis allows the identification of significant predictors of outcome (e.g., disease) (with cut-off points) among factors chosen by the researchers. 

**Figure 2:**
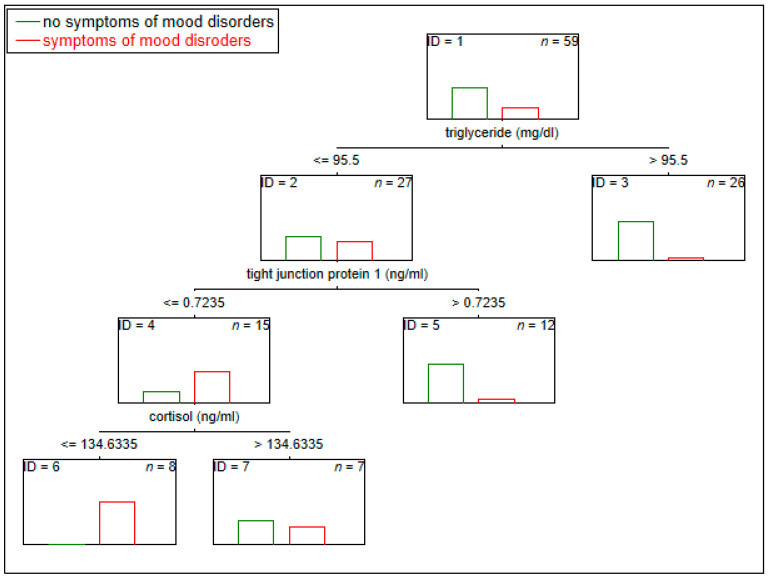
The multi-panel of biomarkers for recognizing mood disorder symptoms.

In the C&RT, we assessed the examined biomarkers as potential biomarkers of the presence of mood symptoms. According to our analysis, the most promising panel of biomarkers of mood symptom assessment included the levels of triglycerides (with a cut-off of ≤95.5 mg/dL), TJP1 (with a cut-off of ≤0.72 ng/mL), and cortisol (with a cut-off of ≤134.63 ng/mL) with an area under the ROC curve of 0.87 (95% CI = 0.752−0.988) (Figure 2).

## 4. Discussion

Our study aimed to assess the consequences of ACEs and experienced stress on depressive symptoms and the role of biological disturbances in this connection. The study’s results suggest that ACEs may exert long-term deleterious effects on mental health and predispose individuals to the development of depression. According to the analysis of the appearance of depressive symptoms, the interaction between experienced stress and earlier unpleasant experiences must occur to develop symptoms. Biological factors could also negatively affect mental health, enhancing the negative impact. The regression analysis confirmed that ACEs and biological factors interplay with psychological stress, leading to the experience of depressive symptoms. 

It should be noted that most (80%) of the examined population had a high severity of stress symptoms, but only one-third of them (27%) had a higher risk of MDD development (based on the BDI scale score). Depression may occur after exposure to psychological stress, but other factors are required to develop symptoms. According to meta-analyses, early traumatized individuals are more sensitized to the effects of stressors. Studies confirm that individuals suffering from MDD report more severe childhood difficulties than healthy individuals [36,37,38]. In a meta-analysis conducted in China, the association between emotional abuse and depression was more substantial than other subtypes of childhood adversity [38]. The meta-analysis results are consistent with our study results. We found that depressive symptoms were strongly related to emotional abuse (R = 0.37) and emotional neglect (R = 0.40). Despite the growing knowledge of ACEs on mental health, prevention and early diagnosis in more vulnerable populations is rarely emphasized. Based on our results, ACEs correspond with some biological factors with a potential role in the development or course of MDD. 

An inverse relationship was found between triglyceride levels and emotional abuse and neglect (R = −0.38, R = −0.33, respectively). The triglyceride concentration was proposed as a biological factor by the C&RT blood-based potential biomarker panel linked to depressive symptoms. A study conducted in Poland found a connection between lower triglyceride and other lipidogram parameters and suicidal thoughts in patients suffering from MDD [39]. According to the cholesterol-serotonin impulsivity model, low lipid levels are related to decreased serotonin and serotonin metabolite activity. Cholesterol is a major component of myelin and myelin pathology in MDD and can be attributed to reduced lipid levels. The interaction between peripheral and central cholesterol levels has been confirmed [40]. However, these findings contradict the meta-analysis in which authors found that first-episode MDD is related to increased triglyceride levels. The mechanisms underlying these changes are still unclear. We did not examine the student population’s total cholesterol or cholesterol fraction levels. It should be noted that higher levels of lipid fractions in MDD patients should also result from an unhealthy lifestyle. The critical elements related to lipid-disturbed metabolism, i.e., food rich in dietary fat and a sedentary lifestyle, have been suggested as a factor responsible for the higher severity of depression [41,42].

According to our results, OCLN was positively associated with physical abuse (R = 0.31). Occludin is a functional component of the tight junctions (TJs), responsible for regulating epithelial proliferation and survival, facilitating viral entry, and organizing specialized epithelial structures. Animal model studies confirm exogenous stressors that engage OCLN in the abovementioned physiological processes to allow cells for protection [43]. Little is known about the role of OCLN in mental health. However, these TJs are proposed as a potential biomarker of blood-brain barrier (BBB) injury [37,38] and early neurological deterioration [44,45]. Higher levels of OCLN have been observed in patients with bipolar disorder, autism spectrum disorder, and schizophrenia compared to healthy individuals [46,47,48]. The dysregulation of BBB TJs observed in patients with psychiatric diseases could be partially the effect of ACEs.

Emotional neglect was related to the intestinal integrity barrier marker LBP (R = 0.38). Childhood trauma and abuse have been shown to be a risk factor for irritable bowel syndrome development [49]. The effects of ACEs on the gut microbiome responsible for maintaining the integrity of intestinal layer mucosa have been shown [50]. LBP reflects microbial translocation and could be responsible for inflammation. Disruption in the pro-/anti-inflammatory state is strongly related to worsening mood symptoms and poorer patient outcomes [51]. LPS may interact with the central nervous system (CNS), causing neuroinflammation and oxidative stress, which can affect the developing symptoms of depression and anxiety. Intraperitoneal administration of LPS induces an inflammatory response and leads to the appearance of depression-like symptoms in animals [52]. However, in our study, another intestinal barrier integrity marker (TJP1, with a cut-off of ≤0.72 ng/mL) was proposed as a marker of depressive symptoms.

The cortisol level (cut-off point of ≤134.63 ng/mL) was a proposed factor to distinguish depressive symptoms and was inversely related to emotional abuse (R = −0.35). Cortisol is the end product of the activation of the HPA axis, essential for an adequate response to psychological stress. Stress is a well-known factor in increasing the risk of psychiatric disorders, including depression. A meta-analysis has shown a sex-specific cortisol stress response with a blunted reaction in women [53]. This finding is in line with our results—most examined students were female. The cortisol response in the luteal phase of a woman’s menstrual cycle is similar to a man’s response. It has been shown that using oral contraceptives (used by 9% of students examined by us) reduces free cortisol levels. Baseline levels of sex hormones could, to some extent, explain sex differences in HPA-axis activity between women and men. A blunted cortisol response is also related to more severe depressive symptoms [53]. A lower cortisol concentration is partly counter-regulated by a higher sensitivity of pro-inflammatory factor secretion to the suppressive effects of glucocorticosteroids (GCs), which has been shown in post-traumatic stress disorder. Nevertheless, compensatory upregulation of GC sensitivity is probably insufficient to protect against the negative impact promoted by inflammation [54].

The most promising factors promoting the recognition of the occurrence of depressive symptoms were triglyceride, TJP1, and cortisol, with an 87% diagnostic ability according to the C&RT classifier system. Biological changes may underlie onset or major depressive disorder onset or worsening of MDD. Studies concerning various blood-based biomarkers, including gastrointestinal, immunology, neurotrophic, hormones, and oxidative stress factors, have been conducted. Despite progress in diagnosis, evidence for leading biological theories for onset and maintenance of MDD is still lacking [55]. Hence, the multi-panel of biomarkers presented here could considerably improve the predictability of the disease [56]. Further studies are necessary to confirm our preliminary results.

Despite the high prevalence of stress symptoms in this student population (78% in the present study), preventive programs are rarely implemented at universities. According to our analysis, the length of education was inversely associated with depressive symptoms, which suggests that adaptation to a new environment can have a negative impact on mental health. A meta-analysis has confirmed that cognitive, behavioural, and mindfulness interventions reduce stress symptoms [57]. Effectiveness has also been confirmed in self-guided management programs, which indicate their role in multi-component interventions for reducing psychological problems among students [58]. More attention should be paid by university communities to implement prevention strategies. In our previous study, a higher stress severity was related to unfavourable changes in body composition. Psychological stress increases vulnerability to somatic complications [59].

Limitations should be considered while interpreting the results of the study. Firstly, the assessment included only 60 participants, mainly women. Additionally, the majority of participants in this study were medical students. The results of our work cannot be generalized to other populations and need confirmation in studies among a group of students of different majors. In further studies with a larger population, the menstrual cycle should be considered a factor affecting biological variables. Genetic analysis was beyond the scope of our examination, and extending the research to include this aspect could be valuable for a better understanding of the interplay between somatic and mental health. An additional investigation by experienced clinical psychologists would promote a better understanding of young people’s behaviours and motives.

## 5. Conclusions

The present study confirms the association between ACEs and depressive symptoms in university students and suggests the importance of psychological stress in developing mood disorders. ACEs could have long-term negative impacts not only on mental but also physical health, reflected by changes in specific physiological indicators. Some of the biological markers could be helpful in the early detection of depressive symptoms in healthy individuals, but their direct effect on MDD development has not been confirmed. Further studies examining the role of biological factors in the aetiology or pathophysiology of MDD among individuals who experienced ACEs are needed.

## Figures and Tables

**Table 1:** Characteristics of the examined population.

Variable		%	*n*
Year of study	I	8	5
	II	10	6
	III	13	8
	IV	22	13
	V	27	16
	VI	20	12
Dietary supplements	Vitamin D	30	18
	Probiotics/prebiotics	5	3
	Hair growth	5	3
	Vitamin B	2	1
	Vitamin C	2	1
	Fish oil	2	1
	Creatine	2	1
Variable		X−/Me	SD/min–max
Age (years)		23	1.9
Education period (years)		16	13–19
PSS-10 (points)		22	3.48
BDI (points)		6	0–26
CTQ (points)		34	25–68

%—percent; N—number; X—mean; Me—median; SD—standard deviation; min—minimum; max—maximum; PSS-10—Perceived Symptoms Scale; BDI—Beck Depression Inventory; and CTQ—Childhood Trauma Questionnaire.

**Table 2:** The predictors of mood disorder symptoms according to regression analysis.

	Model 1		Model 2		Model 3	
Variability	29.23%		26.04%		33.83%	
	β	*p*	β	*p*	β	*p*
CTQ (total score)	0.29	0.028	0.35	0.008	0.32	0.009
PSS-10 (total score)	0.26	0.049	0.30	0.027	0.25	0.04
Education period (years)	−0.26	0.045	−0.23	0.077	−0.32	0.008

β—beta-coefficient; *p*—*p*-value; CTQ—childhood trauma questionnaire; PSS-10—Perceived Stress Scale; Model 1: adjusted for TG and cortisol; Model 2: adjusted for TG; Model 3: adjusted for cortisol.

## Data Availability

Further information is available from the corresponding authors upon reasonable request.
